# Omega-3 Alpha-Linolenic Fatty Acid Affects the Level of Telomere Binding Protein TRF1 in Porcine Skeletal Muscle

**DOI:** 10.3390/ani10061090

**Published:** 2020-06-24

**Authors:** Magdalena Ogłuszka, Marinus F. W. te Pas, Ewa Poławska, Agata Nawrocka, Kamila Stepanow, Mariusz Pierzchała

**Affiliations:** 1 Department of Genomics and Biodiversity, Institute of Genetics and Animal Breeding of the Polish Academy of Sciences, 05-552 Jastrzębiec, Poland; e.polawska@ighz.pl (E.P.); a.nawrocka@ighz.pl (A.N.); k.stepanow@ighz.pl (K.S.); m.pierzchala@ighz.pl (M.P.); 2 Animal Breeding and Genomics, Wageningen UR Livestock Research, 6700AH Wageningen, The Netherlands; marinus.tepas@wur.nl

**Keywords:** pigs, telomere, shelterin, omega-3 fatty acid, TRF1, skeletal muscle, aging

## Abstract

**Simple Summary:**

Polyunsaturated omega-3 fatty acids are nutrients with well-described beneficial effects for human and animal health. However, their role in preventing premature aging and aging-related diseases is not fully understood. The main indicators of biological aging are telomeres. Here, we studied the impact of omega-3 fatty acid contained in linseed oil on telomere biology in porcine muscle. Pigs supplemented with omega-3 fatty acids had lower levels of the TRF1 shelterin protein, which is known to protect telomeric sequences. Additional experiments are needed to better understand the effects of omega-3 fatty acids on telomere biology in muscles, and the effects that may be ascribed to their antioxidant properties.

**Abstract:**

Omega-3 fatty acids are health-promoting nutrients that contribute to the amelioration of age-related diseases. Recent studies have reported the role of these fatty acids in the aging process, explicitly impacting telomere biology. The shelterin protein complex, located at the extremities of chromosomes, ensures telomere protection and length regulation. Here, we analyzed the impact of dietary omega-3 alpha-linolenic fatty acid from linseed oil on skeletal muscle telomere biology using an animal model of female pigs. Fifteen animals were supplemented with linseed oil for nine weeks and an equal number of individuals were fed with a control diet. Linseed-oil-supplemented animals showed an increased level of alpha-linolenic acid in skeletal muscles compared to control animals. There was no difference between groups in the telomere length measured in leukocytes and muscles. However, muscles of the linseed-oil-supplemented pigs showed lower levels of the shelterin TRF1 protein compared to the control group. Our results suggest that omega-3 linolenic acid counteracts the elevation of TRF1 levels, which increase with age and due to the presence of reactive oxygen species in muscle. The observed effect may be due to attenuation of oxidative stress.

## 1. Introduction

The extremities of eukaryotic chromosomes, which are known as telomeres, consist of highly conserved hexanucleotide repeats (TTAGGG) varying in length from 5 to 15 kilobases [1]. Telomeres, together with shelterin proteins, are essential for maintaining genomic stability by protecting the chromosomes from degradation, end to end fusion, and activation of double-strand-break-repair systems. In normal eukaryotic cells, including the stem cells of renewal tissues, telomeres are shortened due to incomplete copying of the 3′ DNA strand extremities during DNA replication [2]. Consequently, cells lose between 50 and 150 bp of the telomere repeats every replication cycle. Telomere shortening is therefore associated with cell division, and has also been found to be associated with various diseases and oxidative stress [3]. Telomere dysfunction is associated with cancer, cardiovascular diseases, premature aging, cognitive decline, and neurodegenerative diseases [4,5,6].

Enzymatic protection mechanisms (telomerase) and protein structures (shelterin) have been associated with telomere maintenance. The telomerase enzyme with reverse transcriptase activity is responsible for the extension of the telomeric sequence. However, nearly all somatic cells do not have telomerase activity [7]. Telomeres are associated with the shelterin protein complex. Shelterin interacts with proteins and signaling complexes to control telomere length. The shelterin complex (telosome) is composed of TRF1 (repeating-binding telomeric factor 1), TRF2 (telomeric repeating-binding factor 2), TIN2 (TRF1- and TRF2-interacting nuclear protein 2), Rap1 (repressor/activator protein 1), TPP1 (also known as adrenocortical dysplasia protein homologue), and POT1 (protection of telomeres 1). TRF1 can co-immunoprecipitate with the protein kinase ATM (ataxia telangiectasia mutated) which acts as a DNA damage sensor [8]. Furthermore, TRF1 and TRF2 function as protein-interaction hubs within the telomere signaling network, interacting directly with the other members of the telosome and with a diverse array of proteins and protein complexes that are involved in the cell cycle and in DNA repair and recombination [8,9]. Recent studies on shelterin proteins have highlighted an equally important role of telomere-bound proteins in disease and aging. Changed expression of TRF1, TRF2, and TIN2 found in human cancers, such as increased expression of TRF1 in chronic lymphocytic leukemia cells [10] and decreased expression in renal cell carcinoma [11], raised the possibility of using these components as potential therapeutic targets for cancer treatment [12]. Abnormalities in TRF1 and TRF2 level were also related to myopathies such as Duchenne muscular dystrophy, dermatomyosis, and polymyositis [13,14]. Nevertheless, the role of shelterin proteins in telomere biology and disease remains poorly understood.

There have been a few reports regarding the effect of omega-3 fatty acids on telomere senescence. In recent years, increasing research interest has been devoted to the influence of omega-3 fatty acid intake on telomere length. Among patients with coronary artery disease, there was an inverse relationship between baseline blood levels of marine omega-3 fatty acids and the rate of telomere shortening [15]. Additionally, O’Callaghan et al. [4] postulated that telomere shortening in patients with mild cognitive impairment might be reduced by omega-3 fatty acid supplementation. 

The mechanism by which polyunsaturated fatty acids (PUFAs) affect telomere functioning is not well-understood. Telomere length is likely regulated by exposure to inflammatory cytokines [16], and Das [17] postulates that this is a mechanism by which omega-3 fatty acids, which are precursors of anti-inflammatory cytokines, can impact telomere length. Alternatively, the effect of omega-3 fatty acids on telomere biology may be mediated through changes in signaling proteins or a reduction of oxidative stress by these fatty acids.

Even less is known about the impact of omega-3 fatty acids on the senescence and telomere biology of muscle tissue. The skeletal muscle is a plastic tissue able to adapt to physical activity and dietary changes. Muscle is a largely post-mitotic tissue; however, muscle satellite cells, quiescent mononucleated myogenic cells, retain proliferative capacity and give rise to regenerated muscle and to more satellite cells [18]. An inverse relationship between age and skeletal muscle telomere length has been reported [19]. Potentially, telomere attrition limits the proliferative capacity of satellite cells that are essential for growth and regeneration [20]. Reaching the critical telomere length may lead to loss of skeletal muscle mass or sarcopenia, and consequently loss of physical performance. Therefore, there is a clear need to identify factors that could counteract muscle telomere attrition.

This study aimed to compare telomere length and shelterin protein levels between groups of pigs with and without omega-3 fatty acid supplementation. In addition, we studied the effect of such supplementation on the fatty acid profile of skeletal muscle and the level of oxidative stress indicators. Because omega-3 fatty acids are antioxidants, we predicted that telomere length would be longer in supplemented groups. 

## 2. Materials and Methods 

### 2.1. Animals 

Female Polish Landrace × Duroc pigs (*n* = 30) were bred at a commercial farm (Drobin, Poland) and fed with a standard diet until pigs reached approximately 60 kg body weight (105 ± 2 days of age). The animals were kept individually in pens equipped with nipple drinkers, on a concrete floor without straw. Pigs received a dry granulated fodder delivered twice a day. Tested animals had the same father and their mothers were sisters. Pigs were then divided into two groups and fed with one of two diets until slaughter. Pigs fed with the control diet received a regular feed mixture (Table 1) containing 25 mg of α-linolenic acid (ALA)/100 g. Pigs in the experimental group received a diet supplemented with linseed oil, which comprised 3% of the total diet. The experimental diet contained 1174 mg of ALA/100 g. The amount of linseed oil was selected based on our previous studies [21], which revealed that approximately 3% of this oil in porcine fodder led to decreased blood lipid indicators and enabled the production of pork with a favorable linolenic acid content. Both diets contained antioxidants—vitamin E and selenium. The diet mixtures were isocaloric (13 MJ EM per kg of the mixture) and balanced according to the amino acid composition. Pigs were delivered to a commercial slaughterhouse (Sierpc, Poland) at least 24 h before slaughter once they reached a body weight of approximately 110 kg (168 ± 2 days of age). Pigs were sacrificed by exsanguination after electrical stunning according to industry standards. Blood was collected during slaughter. Gluteus medius muscle samples were taken immediately after slaughter. Samples were frozen in liquid nitrogen and stored at −80 °C for further analysis. Ethical approval was obtained from the Local Ethics Commission for Experimentation on Animals in Warsaw (No 27/2009). The ethics committee’s permission was issued for a multi-year project.

### 2.2. Gas Chromatography

Quantitative data for the fatty acid profiles in Gluteus medius muscle samples were obtained by gas chromatography using a flame ionization detector (GC–FID) analysis. For each sample, 1 g of the muscle tissue was homogenized and extracted with chloroform–methanol 2:1(v/v), according to Folch et al. (1957). Briefly, tissue samples were homogenized in 5 mL of methanol and 10 mL of chloroform. After filtration through filter paper (Filtrak No. 390), 800 mL of the filtrate was placed in a water bath at 50 °C and evaporated in a stream of nitrogen. The residue was saponified for one hour in a 0.5 M solution of potassium hydroxide in methanol in a water bath at 75 °C. After saponification, samples were esterified with 4% solution of thionyl chloride in methanol, and then the methyl esters were extracted with heptane. Samples were desalted using sodium chloride in order to separate the organic layer. Fatty acids were analyzed using a GC-7890 Agilent gas chromatograph (Agilent Technologies, Inc., Santa Clara, CA, USA) equipped with a 60 m capillary column (Hewlett-Packard-88, Agilent J&W GC Columns, Santa Clara, CA, USA) with a 0.25 mm inner diameter and coating thickness of 0.20 μm. Helium was used as a carrier gas at a flow rate of 50 mL.min^−1^. The injector and detector were both maintained at 260 °C. Column oven temperature was programmed to increase from 140 °C (held for 5 min) at a rate of 4 °C.min^−1^ to 190 °C, and then to 215 °C at a rate of 0.8 °C.min^−1^. Individual fatty acid peaks were identified by comparison with known reference methyl esters (Supelco 37 Component FAME Mix, 47885-U, Sigma-Aldrich Co., Warsaw, Poland) and expressed as a percentage of total fatty acid concentration [22,23].

### 2.3. Telomere Length Measurement 

Nuclear DNA was extracted from the whole blood and muscle samples using a Promega Wizard Genomic DNA Purification Kit (Promega, Warsaw, Poland) following the manufacturer’s protocol. DNA extraction was based on a four-step process. The first step was the lysis of cells and the nuclei. For isolation of DNA from whole blood samples, this step involved lysis of the red blood cells, followed by lysis of the white blood cells and their nuclei. Subsequently, RNA was removed via RNase digestion. The cellular proteins were then removed by a salt-precipitation step. Finally, the genomic DNA was concentrated and desalted by isopropanol precipitation. Nuclear DNA was obtained with good efficiency, and the average ratio of absorbance at 260 nm and 280 nm was 1.9 for blood and 2.0 for muscle samples. Telomere length was measured using the method designed by Cawthon, which is based on real-time PCR [24]. The premise of this method is based on quantification of telomeric DNA in relation to single-copy gene quantity. For this purpose, for each sample, two separate real-time PCR reactions were performed. Here, the single-copy gene was *36B4*, which encodes a ribosomal protein lateral stalk subunit P0. Telomeric and 36B4 primers sequence are shown in Table 2. Each reaction contained 10 µL of 2× LightCycler 480 SYBR Green I Master (Roche, Warsaw, Poland), 200 nM of forward and reverse primers (telomeric or *36B4*), 10 ng of genomic DNA, and water up to the total reaction volume of 20 μL. All real-time PCRs were performed on a Roche LightCycler 96. The cycling conditions for telomeric DNA were 95 °C for 10 min, followed by 40 cycles of 95 °C for 15 s, and 56 °C for 60 s. The cycling condition for *36B4* were 95 °C for 10 min, followed by 40 cycles of 95 °C for 15 s, and 58 °C for 60 s. Standard curves were generated using serial dilutions of a composite sample containing equal parts of DNA from all DNA extracts. Standard curves had an R^2^ > 0.98, and the reaction parameters were as follows: for blood samples: *36B4* reaction: R^2^ = 0.98, PCR efficiency: 2.03; telomere reaction: R^2^ = 0.98, PCR efficiency: 1.95; for muscle samples: *36B4* reaction: R^2^ = 0.98, PCR efficiency: 2.05; telomere reaction: R^2^ = 0.98, PCR efficiency: 1.92. We conducted PCR analysis twice. The inter-plate variabilities were assessed as variations of Ct values for same dilution series in two independent plates. They were highly satisfying (the average inter-assay variations of Ct values for blood samples were: *36B4* reaction: 0.18%, telomere reaction: 0.57%; for muscle samples: *36B4* reaction: 0.37; telomere reaction: 0.98%). Each reaction was conducted in triplicate and data were averaged. PCR product specificity was checked in each case by running a final melting step: 95 °C for 10 s, 65 °C for 60 s, and 97 °C for 1 s in continuous acquisition mode. The Light Cycler U96 Software was used for data analysis. Relative telomere length was measured in accordance with the Cawthon’s telomere measurement method [20]. It was calculated by dividing the telomere PCR product quantity (T) by the reference PCR product quantity of the 36B4 single-copy gene (S). The telomere (T) and single-copy gene (S) quantity were measured with the PCR efficiencies of the reactions taken into account. The PCR efficiencies were calculated based on the slopes of the standard curves, using the formula Efficiency = 10^−1/slope^. Correlation between quantitative variables were assessed using the Spearman rank correlation test.

### 2.4. ELISA Tests 

Protein levels of TRF1, TRF2, TIN2, and ATM were determined using commercial porcine-specific ELISA kits (MyBioSource, San Diego, CA, USA; catalog numbers: MBS104401, MBS7204607, MBS107758 and MBS037213, respectively). The amount of 8-epi-Prostaglandin F2 alpha (8-epi-PGF2α) was determined using a universal reactivity ELISA kit (Elabscience, Houston, TX, USA; catalog number: E-EL-0041). All procedures were performed according to the manufacturer’s protocol. Briefly, homogenates (10 mg of tissue in 100 μL of PBS) and standards were distributed into 96 well plates coated with particular antibodies. Corresponding antibodies labeled with horseradish peroxidase (HRP) were added to each well. Next, a 3,5,3′,5′-tetramethylbenzidine (TMB) substrate solution was added to each well. The TMB substrate becomes blue after catalyzation with the HRP enzyme. The reaction was terminated by the addition of sulfuric acid solution. The colorimetric measurement was carried out using a spectrophotometer (Synergy 4 Hybrid Microplate Reader, BioTek, Winooski, VT, USA) at 450 nm. Results were calculated using standard curves created in individual tests. Unpaired *t*-tests were used to estimate differences among groups. 

### 2.5. Quantitative Real-Time PCR Analysis

mRNA levels of muscle *Fas associated via death domain (FADD*), an oxidative stress indicator, were measured by real-time PCR. Total RNA was isolated from muscle samples using an SV Total RNA Isolation System Promega (Madison, USA) according to the manufacturer’s recommendations. RNA was obtained with good efficiency, and the average ratio of absorbance at 260 nm and 280 nm was 1.95. RNA was reverse-transcribed into cDNA using oligo-dT primers and the Transcriptor First Strand cDNA Synthesis Kit (Roche Applied Science). Expression was normalized to the *glyceraldehyde-3-phosphate dehydrogenase (GAPDH)*. Intron-spanning primers, presented in Table 2, were specifically designed to quantify target mRNA transcripts. All reactions were performed on the Light Cycler 96 (Roche Diagnostics, Mannhein, Germany) using the SYBR Green methodology (Roche Diagnostics). Each reaction contained 10 µL of 2× LightCycler 480 SYBR Green I Master (Roche, Warsaw, Poland), 200 nM of forward and reverse primers, 50 ng of cDNA, and water up to the total reaction volume of 20 μL. Cycling conditions were as follows: 95 °C for 5 min followed by 40 cycles (95 °C for 10 s, 58 °C for 10 s, and 72 °C for 10 s). PCR products’ specificity was checked in each case by running a final melting step: 95 °C for 10 s, 65 °C for 60 s, and 97 °C for 1 s in continuous acquisition mode. The standard curve determined based on a serial dilution of pooled cDNA samples allowed an appropriate dilution of cDNA to be established. Standard curves had an R^2^ > 0.96, and the reaction parameters were as follows: for the *FADD* gene: R^2^ = 0.96, PCR efficiency: 2.02 and for the *GAPDH* gene: R^2^ = 1.00, PCR efficiency: 2.05. We conducted PCR analysis twice. The inter-plate variabilities were assessed as variations of Ct values for same dilution series in two independent plates. They were highly satisfying (the average inter-assay variation of Ct values for the *FADD* reaction was 0.20%, and for the *GAPDH* reaction was 0.19%). Each reaction was conducted in triplicate and data were averaged. The Light Cycler U96 Software was used for data analysis. Based on standard curves, taking into account the efficiency of the reaction, the levels of *FADD* transcript were calculated and further normalized relative to *GAPDH* gene as an endogenous control. Unpaired *t*-tests were used to determine differences among groups.

## 3. Results

### 3.1. Effect of Linseed Oil on the Fatty Acid Composition of Pigs Muscle

To evaluate the association between linseed oil supplementation and tissue levels of omega-3 fatty acids, we used GC to analyze muscle fatty acid profiles. Table 3 shows a nearly 4-fold increase in the muscle tissue level of omega-3 alpha-linolenic acid in pigs receiving the diet supplemented with linseed oil compared to pigs fed the control diet. The level of eicosapentaenoic acid (EPA), product of alpha-linolenic acid elongation and desaturation, also showed a tendency (*p* = 0.06) towards increased levels in the muscle tissues of supplemented animals relative to control ones. Concomitantly, we did not observe any significant changes (*p* > 0.05) in the levels of other fatty acids between these two groups.

### 3.2. Leukocyte and Skeletal Muscle Telomere Length

**Figure 1:**
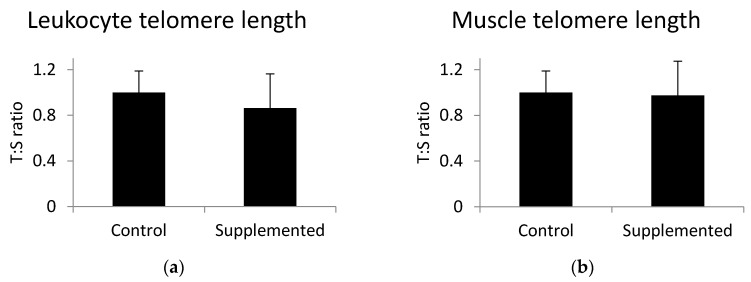
(**a**) Leukocyte and (**b**) gluteus medius muscle telomere length determined using Cawthon’s method. T:S is the ratio of telomere repeat copy number to a single gene copy number. Values are the mean +/− standard deviation. Statistical differences were analyzed by unpaired *t*-test.

To investigate the association between omega-3 fatty acids and telomere length, we analyzed the telomere length in leukocytes and skeletal muscle tissue using PCR. There was no significant difference (*p* > 0.05) in telomere length in either leukocytes (Figure 1a) or skeletal muscle (Figure 1b) between the control group and the group fed the linseed oil-supplemented diet. Muscle telomere length was not correlated with the level of alpha-linolenic acid (r = 0.09; *p* = 0.62). 

### 3.3. Effect of α-Linolenic Acid on the Concentration of Proteins of the Shelterin Complex

**Figure 2:**
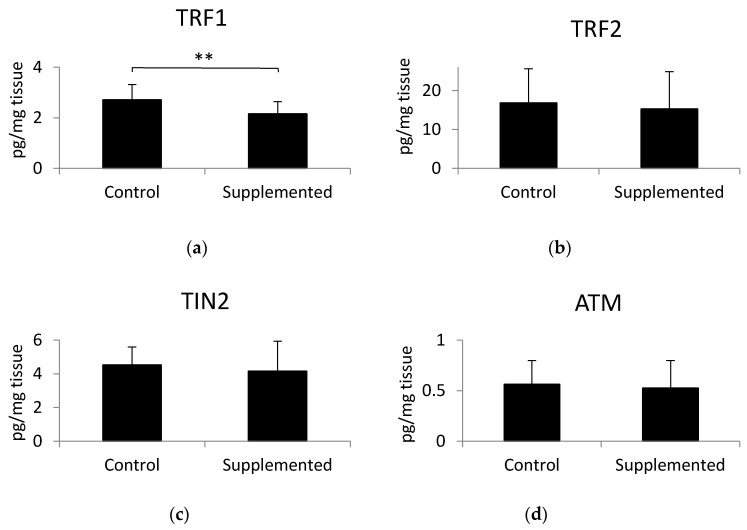
Levels of (**a**) telomere repeat factor- 1 (TRF1), (**b**) telomere repeat factor-2 (TRF2), (**c**) TRF1-interacting protein 2 (TIN2), and (**d**) ATM kinase in muscle samples, determined using ELISA tests. Values are the mean +/− standard deviation. Statistical differences were analyzed using the unpaired *t*-test. Differences of *p* < 0.05 were considered statistically significant, ** *p* < 0.01.

The concentration levels of three proteins of the shelterin complex, TRF1, TRF2, and TIN2, as well as ATM kinase, were analyzed in gluteus medius muscle samples using ELISA tests. Results indicated that a significant decrease of TRF1 protein belonging to the shelterin complex was found in gluteus medius muscles of pigs fed a linseed-oil-supplemented diet compared to the levels observed in pigs fed the control diet (Figure 2a). There were no significant differences in the concentration of TRF2, TIN2, or ATM between the examined groups (Figure 2b–d). The *p*-values calculated by the unpaired *t*-test were as follows: for TRF1 *p* = 0.010, TRF2 *p* = 0.651, TIN2 *p* = 0.496, and ATM *p* = 0.692. Muscle telomere length was not correlated with TRF1 (r = 0.006; *p* = 0.97), TRF2 (r = −0.26; *p* = 0.16), TIN2 (r = −0.08; *p* = 0.69), or ATM level (r = −0.08; *p* = 0.66).

### 3.4. Oxidative Stress Indicators in the Muscle Tissue of Pigs

**Figure 3:**
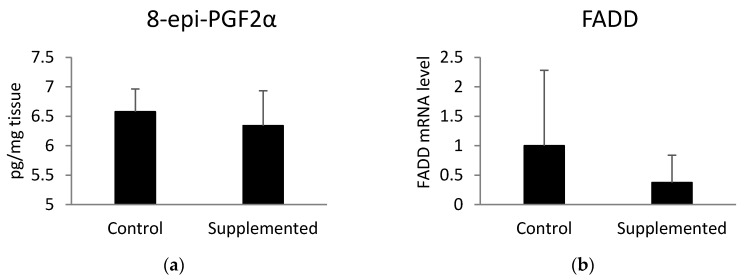
(**a**) Concentration of muscle 8-epi-prostaglandin F2α (8-epi-PGF2α) measured using ELISA test and (**b**) the mRNA level of Fas-associated via death domain (*FADD*) determined by quantitative real-time PCR. Statistical differences were analyzed using unpaired *t*-tests.

In order to examine the level of oxidative stress in pig muscle, we analyzed the concentration of 8-epi-prostaglandin F2α (8-epi-PGF2α). This biomarker of oxidative stress was measured using an ELISA test (Figure 3a) and the mRNA level of *FADD* (Figure 3b), which plays a crucial role in ROS-induced cell death in aging individuals [25]. There was no significant difference in the aforementioned parameters between examined groups. However, the expression of the *FADD* gene (*p* = 0.09) showed a tendency towards downregulation in the muscle tissue of pigs fed the diet supplemented with linseed oil compared to the values observed in the control group. Muscle telomere length was not correlated with the level of 8-epi-PGF2α (r = −0.21; *p* = 0.27) or the expression level of *FADD* (r = −0.17; *p* = 0.38).

## 4. Discussion

Skeletal muscle accounts for approximately 40% of the total body mass and is highly dependent on environmental changes, especially diet and physical activity [26]. Thus, as we expected, the dietary intervention affected the muscle fatty acid composition. The content of α-linolenic acid was significantly higher in the muscle of pigs fed a diet supplemented with linseed oil compared to the content found in pigs receiving the control diet, in line with previous reports [23,27]. The trend towards an increased level of EPA in the supplemented group suggests that elevation of α-linolenic acid level in the diet led to enhancement of its conversion into omega-3 EPA. Such supplementation may impact not only fatty acid composition in skeletal muscle. Studies have shown that increased omega-3 fatty acid content has a positive effect on muscle function and metabolism [26].

Telomere length, especially the leukocyte telomere length, is a proven indicator of biological aging. We studied the effects of dietary omega-3 α-linolenic acid on the regulation of telomere length in young pigs selected for high muscularity and fast growth. Due to selective breeding of pigs, these animals grow extremely fast [28], which is caused by a high rate of cell division [29]. Therefore, it may be expected that the telomeres may be shortened faster than normally expected. However, our study did not reveal significant changes in either leukocyte or muscle telomere length between six month old pigs from the control group and pigs fed the diet with increased content of omega-3 fatty acids, despite the 9 week dietary intervention. Possibly, this period of the dietary intervention was too short to cause detectable changes in telomere length in pigs. Such changes in telomere length can be visible after prolonged supplementation, which has been shown before in human studies for DHA and EPA [4,15]. To our best knowledge, there is no scientific report on the impact of omega-3 fatty acids on porcine telomere biology. However, among patients with coronary artery disease, there was an inverse relationship between baseline blood levels of marine omega-3 fatty acids and the rate of telomere shortening after five years of follow-up [15]. Furthermore, O’Callaghan et al. [4] postulated that telomere shortening might be reduced by omega-3 fatty acid supplementation. This conclusion was drawn based on 6 months of nutritional intervention.

Pigs undergoing intensive farming are highly inbred lines characterized by fast growth rates. Intensive fattening causes several dysfunctions in organisms, such as impaired development of muscles and cardiovascular system, as well as a higher susceptibility to oxidative stress [30]. In these inbred lines, the capillary-to-fiber distance in muscles is too high for proper metabolic removal, and heart strain occurs more often due to a relatively smaller heart in relation to body weight [31]. A deficient oxygen supply may lead to the release of reactive oxygen species (ROS), such as superoxide anions, nitrogen oxide, and hydroxyl radicals, within distinct cell compartments [31]. Various studies have demonstrated that the omega-3 fatty acids contained in linseed oil might efficiently decrease oxidative stress and trigger the expression of enzymes responsible for antioxidant protection in murine muscle cells [32], as well as decreasing mitochondrial ROS production in skeletal muscle of older adults [33]. Furthermore, supplementation of D-galactose-induced aging mice with omega-3 fatty acids led to enhanced activity of superoxide dismutase (SOD), decreased activity of monoamine oxidase, lower concentration of isoprostanes, and decreased level of cerebral lipid peroxidation [34]. Moreover, the concentration of α-linolenic acid in the skeletal muscle was shown to be positively correlated with the activity of GPX1 and SOD2 antioxidant enzymes in these tissues [35]. In our study, the oxidative stress parameter (the expression of the *FADD* gene) exhibited a tendency towards downregulation in the skeletal muscle of linseed-oil-supplemented pigs compared to the control ones.

TRF1 seems to be the negative regulator of telomere length. Higher expression of TRF1 in mouse epidermis leads to telomere shortening [36]. This effect is abolished in the absence of DNA repair endonuclease XPF, suggesting telomere length control through genetic interaction between TRF1 and XPF. Indeed, overexpression of TRF1 caused aberrant sequestration of XPF at telomeric DNA, leading to telomere shortening [36]. On the other hand, TRF1 deletion resulted in early embryonic lethality, but did not affect the telomere length or telomere capping [37]. The level of the TRF1 in muscle tissue was studied by Ludlow et al. [38]. They examined the level of TRF1 protein in the plantaris muscle of 8 week and 1 year old mice. The content of TRF1 significantly increased in the muscle with age [38]. Although the telomere biology of mice and pigs is not identical, because mice show notably longer telomeres due to the lack of telomerase activity repression during embryogenesis [39], the role of TRF1 is comparable [40]. Increased levels of TRF1 may also be a consequence of oxidative stress. Treatment of MGC-803 human gastric cancer cells with arsenic trioxide, which induces reactive oxygen species, caused the upregulation of TRF1 [41]. An increase of TRF1 was also observed in the muscle of patients with Duchenne muscular dystrophy [13], which is characterized by elevated oxidative stress, enhanced ROS [42], and isoprostanes [43]. In our study, we observed a lower level of TRF1 protein in the muscle of pigs fed the linseed-oil-supplemented diet compared to control group pigs. Changes in the concentration of TRF1 may have resulted from the dietary effects of omega-3 fatty acid; however, these changes did not translate into reductions in telomere length, probably because of the short supplementation period (around 9 weeks) used in our experiment. The reason why omega-3 effectively decreased the level of TRF1 in muscles may be ascribed to the antioxidant properties of this fatty acid. Omega-3 fatty acids cause a decrease of oxidative stress and ROS production in the muscle, which possibly counteracts the elevation of TRF1 in the muscle of intensively farmed animals that are exposed to high rates of oxidative stress. 

## 5. Conclusions

To conclude, 9 weeks supplementation with linseed oil, rich in omega-3 ALA, increased the level of ALA and decreased the level of TRF1 in porcine skeletal muscle tissue. Moreover, there was a tendency toward lower levels of *FADD* mRNA, an oxidative stress marker, in the skeletal muscle of pigs supplemented with linseed oil when compared with those fed the control diet. Our results provide evidence that omega-3 fatty acids may be helpful in preventing the TRF1 increase that is usually associated with aging and oxidative stress. Further studies are required to confirm the role of oxidative stress in the described process. Additional studies combining supplementation with omega-3 fatty acids and the use of oxidative stress activators and suppressors may contribute to establishing the exact mechanisms involved in the interaction between omega-3 fatty acids and TRF1 on telomere shortening during the aging process. 

## Figures and Tables

**Table 1:** Composition of pigs’ diets.

Ingredient	Control Diet (%)	Experimental Diet (%)
Rapeseed meal	6.0	7.2
Soybean meal	9.0	9.2
Wheat	54.0	49.6
Barley	28.5	28.5
Linseed oil	0	3.0
Vitamin E	0.1	0.1
Selenium	0.001	0.001
Other	2.4	2.4

**Table 2:** Oligonucleotide primer sequences used for telomere length measurement and quantitative real-time polymerase chain reaction (PCR) analysis of *Fas associated via death domain (FADD)* expression.

Name	Sequence (5′–3′)
TELf	CGG TTT GTT TGG GTT TGG GTT TGG GTT TGG GTT TGG GTT
TELr	GGC TTG CCT TAC CCT TAC CCT TAC CCT TAC CCT TAC CCT
36B4f	TGA AGT GCT TGA CAT CAC CGA GGA
36B4r	CTG CAG ACA TAC GCT GGC AAC ATT
FADDf	GGG CGG GAA GTG TTT GAT T
FADDr	CTC CCT GGC CAA TTC TGT TAT G
GAPDHf	ACT CAC TCT TCT ACC TTT GAT GCT
GAPDHr	TGT TGC TGT AGC CAA ATT CA

TEL: telomere; *36B4: Single copy acidic ribosomal phosphoprotein P0; FADD: Fas associated via death domain; GAPDH: Glyceraldehyde-3-phosphate dehydrogenase.*

**Table 3:** Fatty acid profile [g/100 g Fatty acid methyl ester (FAME)] of the muscle of pigs from control and supplemented groups.

Fatty Acids	Control	Supplemented	*p*-Value
Lauric acid—C12:0	0.10 ± 0.01	0.10 ± 0.01	0.68
Myrystic acid—C14:0	1.36 ± 0.02	1.40 ± 0.02	0.32
Palmitic acid—C16:0	25.58 ± 0.13	25.43 ± 0.14	0.58
Heptadecanoic acid—C17:0	0.10 ± 0.02	0.10 ± 0.02	0.87
Stearic acid—C18:0	14.83 ± 0.21	14.06 ± 0.23	0.09
Arachidic acid—C20:0	0.25 ± 0.06	0.28 ± 0.05	0.77
Palmitoleic acid—C16:1 n-7	2.98 ±0.08	3.27 ± 0.10	0.12
cis-10-Heptadecenoic acid—C17:1 n-7	0.16 ± 0.02	0.14 ± 0.02	0.62
Oleic acid—C18:1 n-9	45.09 ± 0.45	44.41 ± 0.39	0.43
Linoleic acid—C18:2 n-6	7.22 ± 0.34	7.25 ± 0.26	0.96
α-Linolenic acid—C18:3 n-3	0.47 ± 0.03	1.77 ± 0.12	1.01 × 10^−6^ ***
cis-11,14-Eicosadienoic acid—C20:2 n-6	0.06 ± 0.02	0.06 ± 0.02	1.00
Arachidonic acid—C20:4 n-6	0.69 ± 0.14	0.66 ± 0.07	0.91
Eicosapentaenoic acid—C20:5 n-3	0.002 ± 0.001	0.02 ± 0.01	0.06
Others	1.10 ± 0.16	1.04 ± 0.16	0.84

The fatty acid content was analyzed by gas chromatography and is expressed as means and standard error of the means. Comparisons were made between the muscle tissues of pigs fed the diet supplemented with linseed oil versus pigs fed the control diet. Differences of *p* < 0.05 were considered statistically significant, *** *p* < 0.001.
